# Applying Mobile Technology to Sustain Physical Activity After Completion of Cardiac Rehabilitation: Acceptability Study

**DOI:** 10.2196/25356

**Published:** 2021-09-02

**Authors:** Abdelaziz Elnaggar, Julia von Oppenfeld, Mary A Whooley, Stephanie Merek, Linda G Park

**Affiliations:** 1 Department of Community Health Systems School of Nursing University of California San Francisco San Francisco, CA United States; 2 Veterans Affairs Medical Center San Francisco, CA United States; 3 Department of Medicine University of California San Francisco San Francisco, CA United States; 4 Department of Epidemiology & Biostatistics University of California San Francisco San Francisco, CA United States

**Keywords:** physical activity, cardiac rehabilitation, digital health, mobile app, wearable device, mHealth, mobile phone

## Abstract

**Background:**

Many patients do not meet the recommended levels of physical activity after completing a cardiac rehabilitation (CR) program. Wearable activity trackers and mobile phone apps are promising potential self-management tools for maintaining physical activity after CR completion.

**Objective:**

This study aims to evaluate the acceptability of a wearable device, mobile app, and push messages to facilitate physical activity following CR completion.

**Methods:**

We used semistructured interviews to assess the acceptability of various mobile technologies after participation in a pilot randomized controlled trial. Intervention patients in the randomized controlled trial wore the Fitbit Charge 2, used the Movn mobile app, and received push messages on cardiovascular disease prevention and physical activity for over 2 months. We asked 26 intervention group participants for feedback about their experience with the technology and conducted semistructured individual interviews with 7 representative participants. We used thematic analysis to create the main themes from individual interviews.

**Results:**

Our sample included participants with a mean age of 66.7 (SD 8.6) years; 23% (6/26) were female. Overall, there were varying levels of satisfaction with different technology components. There were 7 participants who completed the satisfaction questionnaires and participated in the interviews. The Fitbit and Movn mobile app received high satisfaction scores of 4.86 and 4.5, respectively, whereas push messages had a score of 3.14 out of 5. We identified four main themes through the interviews: technology use increased motivation to be physically active, technology use served as a reminder to be physically active, recommendations for technology to improve user experience, and desire for personal feedback.

**Conclusions:**

By applying a wearable activity tracker, mobile phone app, and push messages, our study showed strong potential for the adoption of new technologies by older adults to maintain physical activity after CR completion. Future research should include a larger sample over a longer period using a mixed methods approach to assess the efficacy of technology use for promoting long-term physical activity behavior in older adults.

## Introduction

### Background

After a major cardiac event, such as myocardial infarction or coronary revascularization, the current class 1A recommendation is to refer patients to cardiac rehabilitation (CR) [1-3]. CR is an important evidence-based exercise and secondary prevention program that reduces mortality and secondary events after cardiac events with the goal of continuing physical activity in patients after program completion [4-7]. However, many studies have shown that patients fail to maintain physical activity after completing CR and often return to a sedentary lifestyle [8,9]. Therefore, more targeted interventions are needed to promote physical activity maintenance after CR completion.

As smartphone ownership increases across age groups [10], mobile health (mHealth) technologies, including text messages or mobile apps, have emerged as a promising interactive intervention to promote self-management of behaviors, such as physical activity [11,12]. Mobile apps coupled with wearable activity trackers are useful tools for the self-management of physical activity. Self-management is achieved through instant visual feedback delivered by the mobile app and stored data on patterns (eg, weekly trends in physical activity) [13,14].

mHealth interventions have shown a range of positive behavioral changes, including increasing self-monitoring and self-care, as well as facilitating peer and social support [15,16]. In addition, mHealth has been used to induce behavioral changes to target self-management of chronic conditions. Physical activity has been one of the main uses of mHealth [17-19] with the advent of wearable devices, however, there is high variability in engagement with health apps (ie, several times a day to once a month). Little is known about the acceptability of these interventions in older populations after completing CR [13,14,20]. To better understand how to maintain behavioral changes after CR, we ascertained patient preferences and experiences after using various mHealth technologies for 2 months following CR completion.

### Objective

The primary aim of this study is to assess the acceptability of using mHealth tools (ie, wearable activity trackers, mobile phone apps, and push through messages) to promote physical activity after completion of CR among older adults. This paper presents a secondary study focused on acceptability that was conducted as part of the primary Mobile4Heart study, a pilot randomized controlled trial (RCT) [21].

## Methods

### Overview and Study Design

**Figure 1:**
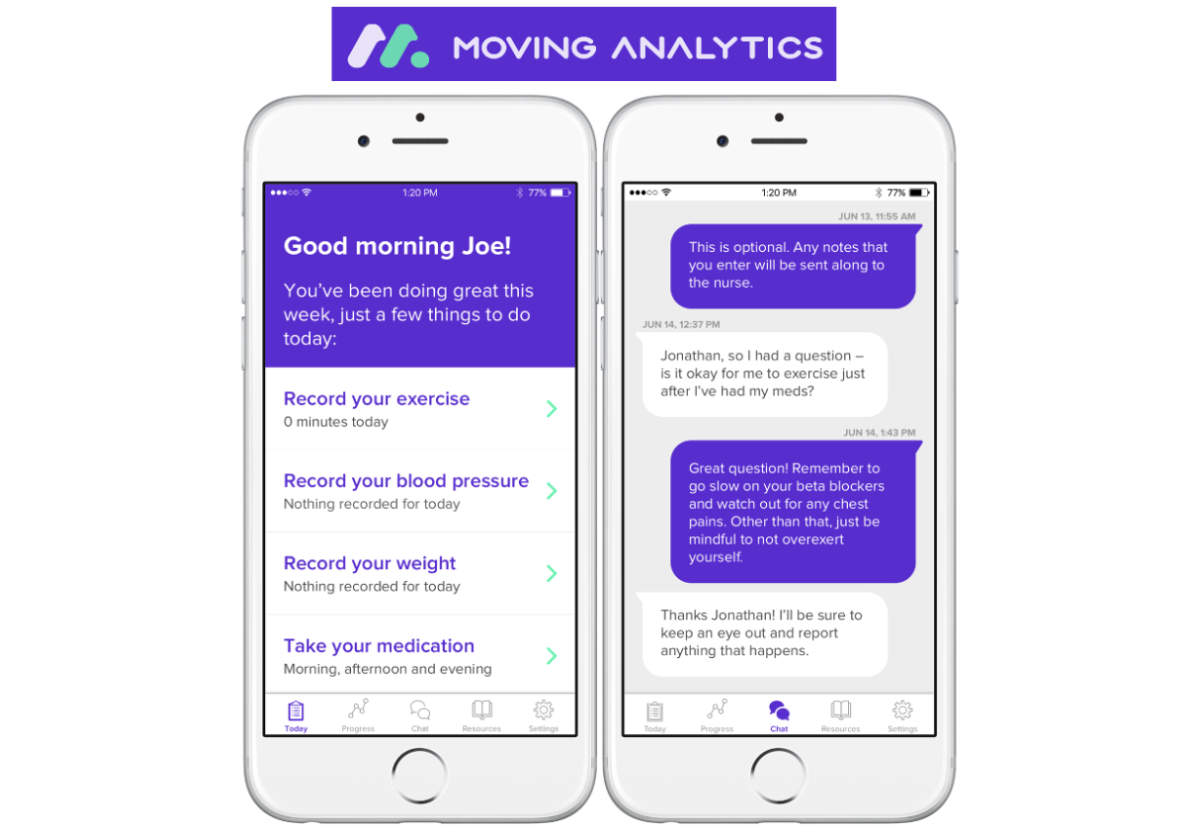
Movn mobile app screen displays.

The parent Mobile4Heart study was a pilot RCT that evaluated group differences in physical activity and exercise capacity after 2 months of using digital health technologies; the results are presented in a separate publication [21]. The 2-month duration was based on the pilot nature of the RCT. Participants in the intervention group were provided with (1) a Fitbit Charge 2 to record their step counts, (2) the Moving Analytics Movn mobile app, which is a smartphone app designed specifically as a telemonitoring tool for CR patients (Figure 1), and (3) push through messages on cardiovascular disease prevention and physical activity delivered through the app. The individuals from the intervention arm of the Mobile4Heart RCT were asked to participate in evaluating the intervention presented in this paper.

This study presents a secondary study using two separate approaches to examine acceptability. We asked the intervention group participants to provide feedback about their experience with the technology (n=26) and conducted semistructured individual interviews with 7 representative participants. During the individual interviews, we used a semistructured interview guide that included a quantitative scale on satisfaction with the different technology components that allowed for an open-ended approach to ask and respond to questions for more substantial feedback on their responses (Multimedia Appendix 1).

We used thematic analysis with an emphasis on the acceptability of the technology. The interviews were transcribed verbatim, and 2 trained researchers independently reviewed and coded the interview transcripts and applied deductive codes developed from the interview guide domains. Through an iterative process and constant comparative approach, we finalized the coding scheme, refined themes, and identified patterns and relationships among the qualitative data.

Mobile4Heart was approved by the institutional review board at the medical center for recruitment as well as by the academic institution that sponsored the study (ClinicalTrials.gov NCT03446313).

### Participants and Setting

Participants were enrolled from 3 community CR centers in Northern California between February 2018 and January 2019. Eligibility criteria for participation included the ability to speak English, age >18 years, and actively participating in CR because of a previous cardiac event that qualified the patient for CR. After meeting the primary inclusion criteria, participants were screened for cognitive impairment using the Mini-Cog test [22-24]. Exclusion criteria included inability to access a smartphone and unstable clinical conditions (eg, unstable arrhythmias, uncontrolled hypertension, active infection, and second- or third-degree heart block).

### Recruitment and Procedures

#### Overview

Full procedure details related to the Mobile4Heart study have been published elsewhere [25]. Briefly, enrollment occurred by meeting participants within 2 weeks after completion of CR, and participation started immediately after the first baseline meeting. All participants provided written informed consent before participation. Sociodemographic characteristics and self-reported physical activity were collected. For this study, all intervention participants were asked about their general experience with the three digital intervention technologies using open-ended questions at the completion of the intervention period. In addition, a convenience sample of 7 individuals participated in semistructured individual interviews that lasted between 45 and 60 minutes, with the completion of a questionnaire about their experiences using the mHealth technologies (Multimedia Appendix 2). We sought to include a representative sample of individuals with varying levels of engagement with the intervention. The interviews were designed to address the perceptions and experiences of participants with the three technologies used for the intervention, facilitators and barriers of use, the impact of these interventions on their future physical activity, and suggestions for improving these interventions. All interviews were conducted in person in a private conference room at the medical center. Participants first completed a Likert scale questionnaire. The interviewer then used open-ended questions to prompt additional inputs based on the responses from the questionnaire. Furthermore, 2 study staff members conducted the interviews, including the principal investigator. The interviews were audio and digitally recorded and transcribed verbatim as raw data for analysis. All participants received separate compensation for participation in the clinical trial, but the 7 participants who completed the interviews received an additional US $25 gift card as compensation for their time.

#### Mobile Phone App

The study staff downloaded the Moving Analytics (Movn) and Fitbit mobile apps on their smartphones (Figure 1). Generic emails and study participation numbers were generated by the study staff to register participants on both apps to protect participants’ data. Both apps were synced wirelessly with the participants’ mobile phones to view step count and physical activity data. In addition, Fitbit data were also synced to Fitabase [26], which is a comprehensive data management platform designed to store Fitbit data in cloud format. The study staff demonstrated both apps to participants and asked them to navigate through both apps. The study staff ensured that the participants were comfortable using the basic functions and features of the Fitbit device and Movn app. The Fitbit device tracked step count and some aerobic exercises such as running and using an elliptical machine; however, only step count was used to measure physical activity in this study. The Movn app was used to track daily weight, blood pressure, heart rate, and medication use and allowed participants to record any physical activity not captured by the Fitbit device (eg, swimming or weightlifting). In addition, the Movn app allowed participants to report any cardiovascular symptoms. The study staff triaged all participants’ symptom entries once a day. If a participant recorded shortness of breath or chest pain, a message prompted participants to immediately call 911 through a button on the app.

#### Wearable Activity Tracker

Participants were provided with the Fitbit Charge 2 to wear upon enrollment during all waking hours for the 2-month study period. Participants were instructed to remove the device when showering and swimming, as the devices were not waterproof. The study staff assisted participants with the basic functions and features of both the device and the app, which included syncing the device with the mobile app and charging the device. Fitbit Charge 2 is a medium-sized wrist-worn activity tracking device that collects real time data about physical activity with a small monitor that provides instant visual feedback to the user. Fitbit devices use a 3-axis accelerometer to translate movement into digital measurements of body movements, frequency, duration, and intensity, and pattern of movement to determine the number of steps taken and distance traveled [27]. In addition, it measures energy expenditure (calories burned) and sleep quality. However, only step count data were collected for this study. This device was chosen for the following reasons: (1) convenience to the participants as it required charging only once every 5 days, (2) data storage for up to 7 days on the device, and once synced with Fitabase, the research team was able to view participants’ performance, (3) the ability to create generic accounts without breaching participants’ privacy, if desired, and (4) the relatively low cost of the device. 

#### Push Through Messages

On the basis of the American Heart Association Simple 7 principles [28], a bank of messages was created that included suggestions on promoting participants to engage in physical activity, healthy nutritional habits, and medication tracking (Multimedia Appendix 1). Some of these messages were one-way; however, most of the messages were two-way, which allowed participants to respond to ensure their active engagement. Through the Movn app, the study staff sent these push through messages three times per week on random weekdays between 9 AM and 6 PM, providing positive feedback and additional motivation for physical activity. This feature also allowed the study staff to craft a personalized text for each participant. Thus, this created an additional communication channel between the study staff and participants to follow up on their progression and to answer any technical questions.

### Data Analysis

The 7 participants who participated in the semistructured interviews were asked to rate their satisfaction regarding the different technologies used in the study on a 1- to 5-point Likert scale (5 being the highest). The scores from the satisfaction survey were presented quantitatively as means. For the interviews, 2 study staff independently coded and analyzed the transcripts using thematic analysis to identify themes and subthemes. Through an iterative process and constant comparative approach, we finalized the coding scheme, refined themes, and identified patterns and relationships among the qualitative data. We discussed the findings after independent coding was completed, and the principal investigator resolved any inconsistencies or discrepancies. Emerging codes from the interviews were used to identify the themes of participants’ acceptability of the wearable device, push through messages, and smartphone apps.

## Results

### Participant Characteristics

A total of 32 participants from the intervention arm of the Mobile4Heart study were eligible to provide feedback on the intervention. Out of the 32 participants, 6 intervention patients were excluded for the following reasons: 1 participant was lost to follow-up, 1 was diagnosed with terminal cancer, 3 failed primary screening, and 1 had a broken toe and was unable to finish the study (Multimedia Appendix 3 shows the CONSORT [Consolidated Standards of Reporting Trials] diagram). Baseline characteristics of the enrolled patients are shown in Table 1. The mean age of participants was 66.7 years (SD 8.6). There were 77% (20/26) male participants and 23% (6/26) female participants, and 73% (19/26) self-identified as White individuals.

**Table 1:** Baseline sociodemographic data (N=26).

Characteristics			All intervention participants (n=26)		Questionnaires and interview participants (n=7)
Age (years), mean (SD)			66.7 (8.6)		64.4 (7.7)
Female, n (%)			6 (23)		2 (29)
**Race or ethnicity, n (%)**					
	Hispanic, Latino, or Latina	1 (4)		0 (0)	
	White	19 (73)		5 (71)	
Married, n (%)			23 (88)		7 (100)
Employed, n (%)			10 (38)		2 (29)
College graduate, n (%)			18 (69)		6 (86)
**Causes for enrollment in cardiac rehabilitation, n (%)**					
	Ischemic heart disease (no)	19 (73)		4 (57)	
	Heart failure (no)	4 (15)		2 (29)	
	Valvular heart disease (no)	2 (8)		1 (14)	
	Structural heart disease (no)	1 (4)		N/A^a^	

^a^N/A: not applicable.

### Findings

#### Overview

There were overall high satisfaction scores for the Fitbit wearable device and Movn mobile app but lower satisfaction scores with the push through messaging feature, as shown in Multimedia Appendix 4.

Participants’ feedback and interviews about their experiences with digital technology yielded four major themes (Textbox 1). Two themes focused broadly on positive experiences with these interventions, whereas the other two themes focused on the limitations and needed improvements.

**Cited themes from qualitative interviews.**

**Themes**

- Technology use increased motivation for physical activity
- Technology use served as a reminder to remain physically active
- Recommendations for technology to improve user experience
- Desire for personal feedback

#### Theme 1: Technology Use Increased Motivation to Be Physically Active

There was a general consensus among all participants that digital technology has robust potential to promote physical activity as it provides a sense of continuity to CR by providing motivation. Using various digital technologies was a key facilitator for increasing motivation. The mean age of the participants was approximately 64 years (SD 7.7), and the general consensus among these older adults was that both apps provided a user-friendly layout:

...it made me feel as if it was an extension of CR.

Participants reported wearing the device on the wrist was a motivational intervention by itself. In addition, daily step feedback through the device as well as the app provided a sense of commitment to complete the daily target for step counts. Participants enjoyed the Fitbit features and functions that enabled them to self-monitor and obtain insight on the distance walked through the number of steps:

Motivated me to walk more and reach the 10,000 steps goal.

This provided a sense of enjoyment in tracking the number of steps and distance walked throughout the day. When one of the participants was asked if the use of technology helped him stay motivated, his response was as follows:

Absolutely! Very necessary for insight.

In addition, the vibrating function of the device when the daily step goal was reached provided additional enforcement of positive physical activity behavior:

Furthered commitment to exercise, incentivized to do better.

Both the Movn and Fitbit apps provided visual feedback about the progress of each participant by viewing weekly steps in a chart review. Many participants highlighted the ease of use and interpretation of the data through both apps:

...and for someone with limited knowledge in technology like me, Fitbit was encouraging for me to keep moving.

Participants emphasized the ability to not only reflect on their daily steps’ progression through charts on their step counts but also to set a new personal target to achieve every day. This allowed an increase in participants’ awareness of their physical activity levels and the progress they achieved. In addition, some participants enjoyed the other features within both apps about heart rate and sleeping performance, which provided some information about their overall physical activity performance as well as their recovery:

Feedback about different health aspects like sleeping and food intake are information to know about myself.

Participants commented on the benefit of receiving push through messages from the study staff through the app throughout the week at random times as a motivational tool to remain physically active, knowing that the study staff is updated with their physical activity status:

Just the fact knowing you guys [study staff] are watching my numbers motivated me to walk more.

In addition, the messages incentivized some participants to perform different exercises other than walking or running. Participants commented that these messages provided some physical activity hints and motivations to set a new personal goal:

The messages gave me some hints and good advice, like stretching.

#### Theme 2: Technology Use Served as a Reminder to Be Physically Active

Participants’ comments about the use of technology were mainly for increasing motivation and a reminder to maintain physical activity. They also enjoyed the Fitbit features and functions that enabled them to self-monitor and obtain insight on the distance walked through the number of steps. The visual display of the device was a sufficient reminder for some participants to remain active. In addition, the device had a vibrating function as a reminder to move in case of inactivity for over 2 hours:

The device gives a nudge every while, which is a good reminder to go out and walk.

Participants attributed their self-awareness of their physical activity through immediate feedback about the number of steps walked during the day as a contributing factor to their behavioral change:

Yes, it was a visual reminder, allows me to track something while I’m walking.

The Movn app also sent a notification reminder around the end of the day to submit any additional workout activities that were not captured throughout the day by the Fitbit device, which had an additional reinforcement effect. Furthermore, the app provided a platform for participants to upload different health measures, such as blood pressure and glucose. These measures were not recorded for this study, but participants commented that it was a convenient tool to track all their measurements in one place. The Movn app also enabled participants to report any cardiac symptoms related to their condition, which would alert the study staff instantly and was triaged by a health professional daily:

Push notification [from the app] throughout the day was helpful to remind me to remain active.

Although patients were instructed to use the Fitbit wearable device to track step counts and the Fitbit app to download steps, some participants chose to explore the other features on the Fitbit mobile app as well. Some of them mentioned enjoying the social interaction feature in the Fitbit app, which was the requested feature of the Movn app to interact with their CR peers. Both apps provided different notification reminders throughout the day.

#### Theme 3: Recommendations for Technology to Improve User Experience

A number of obstacles and barriers were reported by the participants regarding each type of intervention technology, with some suggestions for development and improvement. A common theme was the complaint about the Fitbit device being only water-resistant (not waterproof), which limited the physical activities that could be captured by the wearable device.

In addition, an important comment that may be relevant for many middle-aged to older adults was regarding the size of the text on the wearable device’s screen. Having an accompanying website portal is helpful in providing another way to view data, as Fitbit already provides:

...needs larger print on device. Too small, can’t read that thing.

The limited sensitivity of the heart rate sensor was spotted by multiple participants, as the device takes a few minutes to detect a consistent heart rate change during the workout; therefore, some exercises such as weightlifting or yoga were not accurately recorded:

The heart rate monitor takes about 10 minutes to capture actual heart rate change while working-out.

Doesn’t capture different activities I do in the gym, like weightlifting.

The Movn app showed the progress of each participant over time; however, this feature was only accessible to the research staff, not the participant:

...I stopped using it. I would check the other app (Fitbit) instead because I wanted to see my progress.

Push through messages were sent through the mobile app and were viewable as a notification message on the participant’s phone. Most of the participants did not report any technical issues with the push through messages, presumably because they were accustomed to viewing text messages on their phones. However, some participants reported an inability to read the messages at the beginning of the study as the notification feature was disabled on their phones.

Overall, there were varying levels of acceptance of the Fitbit device; some participants chose to keep the Fitbit (n=8) at the end of the study, whereas others returned them in lieu of US $100 in gift card incentives (n=12). Some sought to purchase more advanced tracking devices (n=2), whereas others already owned a wearable device (n=4).

#### Theme 4: Desire for Personal Feedback

A number of technical challenges and difficulties were reported by the participants regarding each app. Some participants reported that the inability to adjust the targeted number of steps per day because of physical challenges, such as osteoarthritis, was frustrating:

...my knees hurt; I can’t walk 10,000 steps!

Some participants had some barriers with the Movn app features. These were related to the lack of knowledge and understanding about how to use the app, limited number of physical activity progression charts, and insufficient individual feedback or goal setting for each participant:

[Need] more communication, more hand-holding for less tech-savvy people like me.

Doesn’t give feedback, doesn’t provide me with any chart about my progress.

I can’t set my own goals, want to see progress graphs for a longer duration.

Although the Movn app had different educational materials related to cardiovascular disease health, many participants mentioned the need for additional general health and nutritional advice within the app itself:

Would like some nutritional guidance and more health details, like food calories and fat burning.

Although push through messages from the study staff to the participants through the Movn app were appreciated by many participants, there was consistent feedback from the majority of participants about the need for more personalized messages. Many participants felt that the messages were automated and not customized for each participant’s physical activity step counts and personal goals. Subsequently, some participants lost interest in responding to the two-way messages, as they were either too obvious or not personalized:

The texts need to be more personal with some interaction.

The messages were not helpful, nor motivating. Need more specific input and interaction.

It was too obvious; I didn’t know what to respond to you.

## Discussion

### Principal Findings

In this study, we evaluated patients’ perceptions of the acceptability of mHealth interventions to improve physical activity maintenance after completing a CR program. In general, participants found the Fitbit devices easy to use and useful to self-monitor activity and progress on a daily basis. They also reported the ability to use mobile phone apps to track their physical activity progress, which consequently maintains and improves their physical fitness. The push through messages had an additional motivational effect as a reminder to remain active. These three interventions increased participants’ awareness and self-management of their activity levels. Participants’ long-term use of these technologies remains encouraging as some participants chose to keep the Fitbit at the end of the study. Some chose to purchase tracking devices that were more advanced or already owned a wearable device. Some of the participants who returned the devices preferred not to wear an activity tracking device on their wrist.

We also sought to assess the acceptability of mobile apps among an older population. Although we did not instruct participants to use the Fitbit app except to download their step counts, many chose to explore and subsequently use the Fitbit app. The use of the Fitbit app and Movn app was not equal for all participants, and toward the end of the study period, many participants reported using one app while rarely using the other. This highlights that although participants liked tracking their physical activity, entering their additional workout data into the app was cumbersome to some of them. Many participants expressed their desire for a device that captured all workouts that synchronized automatically with their phones without having to manually enter their workout data. In previous studies that used Fitbit devices with a coaching app, researchers found that participants frequently used both the Fitbit app and the intervention app, but several preferred the features and usability of the Fitbit app [29]. Several researchers have addressed this issue by integrating the features of commercially available apps with a second app and pulling data from one app into the other, hence making the intervention more convenient for participants by using only one app [30-32].

Another important objective of this acceptability study was to determine the impact of push through messages on increasing physical activity. The push messages had the lowest satisfaction scores compared with the other technologies, indicating that improvement is needed in this area. The interviews elucidated the need for more personalized and tailored messages that responded to the physical activity that the participants were engaging in. Some responded negatively and ignored the messages when they thought they were automated. This lack of personalization could potentially harm the relationship between the participant and future clinical providers if expectations are not clearly explained upfront (ie, automated vs personalized or a combination). We provided a combination of messages delivered from our bank of messages and personalized messages. On the contrary, other participants reported having a feeling of assurance that the study staff actively monitored their progression, which helped reinforce the participants’ sense of commitment to remain physically active. This finding is consistent with other studies that consider text message interventions to be effective for improving physical activity and significantly increasing the number of steps per day after the intervention [33]. Using all these interventions together provided insights into how this older population could engage with mobile technology to maintain long-term physical activity after CR. As individual needs should be of primary importance, especially when using health-related apps [34-36], tailoring push through messages could easily supplement an intervention that deploys an activity tracking device.

### Comparison With Prior Work

There is currently a dearth of research examining the acceptability of mHealth technologies by older adults after the completion of CR. However, results from several studies indicate that mobile phone apps and text messages are viewed favorably by this age population with important facilitators of ongoing use, such as ease of use and rapid accessibility [37-41]. The findings of this study were similar to those of other studies, particularly the ease of navigating through mobile phone apps [37,40]. However, some participants expressed difficulties with the Fitbit device, including charging issues, limited use because of the need to take the device off while showering, and the device’s inability to capture different types of physical activity and sports. Other challenges involved a lack of understanding of the various features of both apps, suggesting the need for technology training geared toward this age group regarding app style and layout [42-45].

Previous studies have indicated that data security and privacy may be barriers to participant engagement with mobile interventions, particularly with regard to older adults [46,47]. In addition, there are concerns among this group regarding the lack of regulatory oversight [48]. In this study, however, we found that participants expected to share their data on the app as they were aware that the research team at the other end was monitoring their progress and actively interacting with them, which motivated them to keep using the app compared with machine-operated apps [49]. This willingness to engage with the technology and confidence in the research or clinical team has the potential to increase participant commitment to an active lifestyle, as the presence or absence of patient trust affects health outcomes and adherence [50-52]. Many commercially available mHealth apps have substandard privacy and security protection for users [53-55]. It is imperative that researchers vet the apps used in mHealth interventions to maintain this trust. In addition, clearly explaining and demonstrating security settings to participants may help alleviate safety and privacy concerns encountered in future research.

Some participants expressed their interest in sharing their daily steps progression with other participants they met in their CR program to create a sense of friendly competition with each other. Researchers found that users of a social networking app reported that social comparison motivated physical activity, particularly when compared with higher performers [56]. This highlights the need for further research on the benefits of social engagement with peer participants and peer-to-peer support. Adding a social component to future interventions may help with accountability and decrease barriers to physical activity. Providing a supportive web-based community for users to share tips, encouragement, and even engage in small contests with other users could add to the overall participant experience and increase commitment to physical activity after CR.

### Limitations and Strengths

Several limitations of our pilot study should be noted. We had a small sample size with 7 interviews; however, we believe we achieved data saturation on our topic of acceptability with technology to promote physical activity after CR. Our sample mostly comprised White individuals and men who owned smartphones; thus, our findings have limited representation of other diverse racial groups and women. In addition, we recruited all participants from a single institution in Northern California; therefore, our sample may not represent a broader and older population. Although this study provides insight into the initial experience of older adults using mobile technology over 2 months, long-term behavioral changes are unknown.

Despite these limitations, our study provides important insights into the integration of technology-supported care for patients who often fail to maintain regular physical activity after CR and relapse to a sedentary lifestyle that predisposes them to a secondary cardiac event. Our study confirmed previous research that supports the potential impact of a system that provides reminders and motivation to promote self-care behaviors [57,58]. Wearing the activity tracking device provided repeated reminders by showing the number of steps while also nudging the participant to walk after 2 hours of inactivity. In addition, the two-way push through messages provided by the study staff provided additional active reminders. The impact of this combination of tools was shown to be promising in promoting more step counts among intervention patients compared with the control group in our parent study [21], whereas this study provided the acceptability data.

### Conclusions

Our mHealth intervention shows high acceptability of mHealth technologies to promote physical activity among older adults after CR. Participants’ expectations for using mHealth technology included tracking several health metrics, monitoring personal progress, and personalized communication with the research staff. These results provide promising preliminary groundwork for a community-based physical activity program after CR that is supported by secure mHealth technology to provide personalized feedback and social support.

## Appendix

Multimedia Appendix 1 Push through messages bank.

Multimedia Appendix 2 Interview question and satisfaction scale.

Multimedia Appendix 3 CONSORT (Consolidated Standards of Reporting Trials) diagram of Mobile4Heart participants.

Multimedia Appendix 4 Satisfaction scores with technology use in 7 participants.

## Abbreviations

CONSORT: Consolidated Standards of Reporting Trials
CR: cardiac rehabilitation
mHealth: mobile health
RCT: randomized controlled trial
