# Supplementary material for: Applying Mobile Technology to Sustain Physical Activity After Completion of Cardiac Rehabilitation: Acceptability Study
Source: JMIR Hum Factors. 2021 Sep 2;8(3):e25356. doi: 10.2196/25356 (PMC8446842; doi:10.2196/25356)
Supplement: Multimedia Appendix 1 [file humanfactors_v8i3e25356_app1.docx]

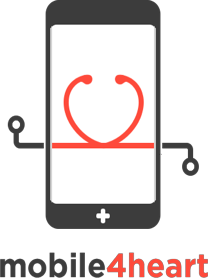
 **Bank of Text Messages for Mobile4Heart**

**8 weeks x 2.5 (2-3 TM/week) = 20**

**1- First message after enrollment:**

- Thank you for being a part of the Mobile4Heart study! We commend you for your interest in taking care of your heart!

**2- Second message after cardiac rehabilitation completion:**

- You are a graduate of cardiac rehab… congratulations on making a great decision to focus on your health!

**3- Throughout the study period (variable messages):**

- Thank you for being a part of the Mobile4Heart study! We commend you for your interest in taking care of your heart!
- You are a graduate of cardiac rehab… congratulations on making a great decision to focus on your health!
- Did you know that sitting less is directly linked to lowering your risk of another heart event?
- Taking the stairs instead of an elevator can be a great way to strengthen muscles and get more exercise
- Try simple ways to get more exercise - work outside, park your car further away, walk your dog longer
- Did you know that just 30 minutes of brisk exercise can improve your mood?
- Have you tried exercising with a friend or calling someone far away to make it more enjoyable?
- Have fun! Exercise doesn't have to be a chore. Think of activities you enjoy and make a plan to do them.
- Remember to stretch before and after exercise to avoid injury.
- Exercise and weight setbacks are normal and often temporary. Stick with your exercise and keep working at it!
- Try something new like yoga, dancing, or gardening to prevent getting bored with your routine.
- You did a great job in cardiac rehab. Talk with someone if you are having a hard time sticking to your goals.
- Are you tracking your success with exercise? Forming habits now will help you succeed in an exercise routine.
- Choose an activity you enjoy - walking, swimming, and riding a bike are some good ways to stay active.
- Exercise makes your heart work better. We hope you are proud of your success!
- Small amounts of walking add up. Instead of parking close to a store's entrance, park farther away.
- Need extra motivation? Find others who you can exercise with.
- Have you tried listening to fun music while you exercise? It can make a huge difference in your mood!
- Developing habits around exercise paves the way for success. Keep exercising regularly and you will see positive results.
- Don't have time for a 30-minute workout? Break it up throughout the day - 10 min 3 times a day works just as well.
- Routines can become boring. Try mixing up the type of exercise, route you walk, etc. to keep yourself engaged and having fun!
- If you have been sitting for more than an hour, consider getting up and moving to reap many health benefits
- If you watch TV, you can exercise or stretch while doing so. Try keeping small weights and resistance bands close by!
- Do you have an exercise plan for when the weather is bad? You can exercise at home or walk at the mall.
- Do you need more energy in the day? Try going for a short walk to get some fresh air.

**4- Last message:**

- Thank you again for coming a part of the Mobile4Heart study. We wish you a lifetime of health and happiness!
