# Supplementary material for: Applying Mobile Technology to Sustain Physical Activity After Completion of Cardiac Rehabilitation: Acceptability Study
Source: JMIR Hum Factors. 2021 Sep 2;8(3):e25356. doi: 10.2196/25356 (PMC8446842; doi:10.2196/25356)
Supplement: Multimedia Appendix 2 [file humanfactors_v8i3e25356_app2.docx]

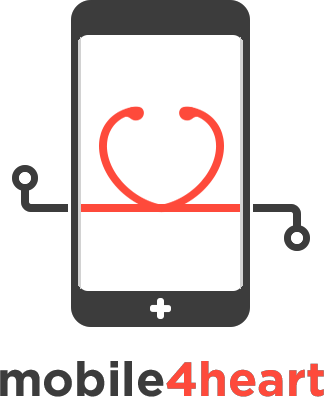
**Process Evaluation for Mobile4Heart:**

**Semi-Structured Individual Interview Guide**

HOW WOULD YOU RATE YOUR…?

- 1. **Overall experience with participating in the Mobile4Heart research study over 2 months?**

1-highly unsatisfied, 2-unsatisfied, 3-neutral, 4-satisfied, 5-highly satisfied

- 1. **Experience working with research assistant**

1-highly unsatisfied, 2-unsatisfied, 3-neutral, 4-satisfied, 5-highly satisfied

- 1. **Enrollment process – 6-minute walk, questionnaires, device set-up**

1-highly unsatisfied, 2-unsatisfied, 3-neutral, 4-satisfied, 5-highly satisfied

FITBIT DEVICE AND APP

- 1. **Overall satisfaction with using the Fitbit wearable device? (overall experience, technical difficulties, ease of use)**

1-highly unsatisfied, 2-unsatisfied, 3-neutral, 4-satisfied, 5-highly satisfied

- 1. **Experience with using the Fitbit wearable device? (technical difficulties, ease of use)**

1-highly unsatisfied, 2-unsatisfied, 3-neutral, 4-satisfied, 5-highly satisfied

- 1. **How often did you interact with the Fitbit wearable device? (minutes/day or times/week)** _________
  2. **Overall satisfaction with using the Fitbit mobile app (overall experience, technical difficulties, ease of use)**

1-highly unsatisfied, 2-unsatisfied, 3-neutral, 4-satisfied, 5-highly satisfied

- 1. **Experience with using the Fitbit mobile app? (technical difficulties, ease of use)**

1-highly unsatisfied, 2-unsatisfied, 3-neutral, 4-satisfied, 5-highly satisfied

- 1. **How often did you interact with the Fitbit mobile app? (minutes/day or times/week) ____________**

MOVN APP

- 1. **Overall satisfaction with using the MOVN app? (overall experience, technical difficulties, ease of use)**

1-highly unsatisfied, 2-unsatisfied, 3-neutral, 4-satisfied, 5-highly satisfied

- 1. **How often did you interact with the MOVN app? ___________**
  2. **Did you think there was any redundancy between the two apps? _________**

TEXT MESSAGES

- 1. **How satisfied were you with the text messages?**

1-highly unsatisfied, 2-unsatisfied, 3-neutral, 4-satisfied, 5-highly satisfied

- 1. **Do you have any recommendations for text messages? ___________**

GENERAL

1. **Would you recommend your friend/family member to participate if he/she was eligible? Why or why not? ___________**
2. **On a scale of 1-10, how likely are you to recommend this study? _________**
3. **What recommendations do you have to improve the research study?**

**___________**

1. **How many minutes per day did you spend participating in the study? ___________**
2. **How do you think participation in the study influenced your exercise and activities after completing cardiac rehab? ___________**
