# Supplementary figures and images for: Applying Mobile Technology to Sustain Physical Activity After Completion of Cardiac Rehabilitation: Acceptability Study
Source: JMIR Hum Factors. 2021 Sep 2;8(3):e25356. doi: 10.2196/25356 (PMC8446842; doi:10.2196/25356)

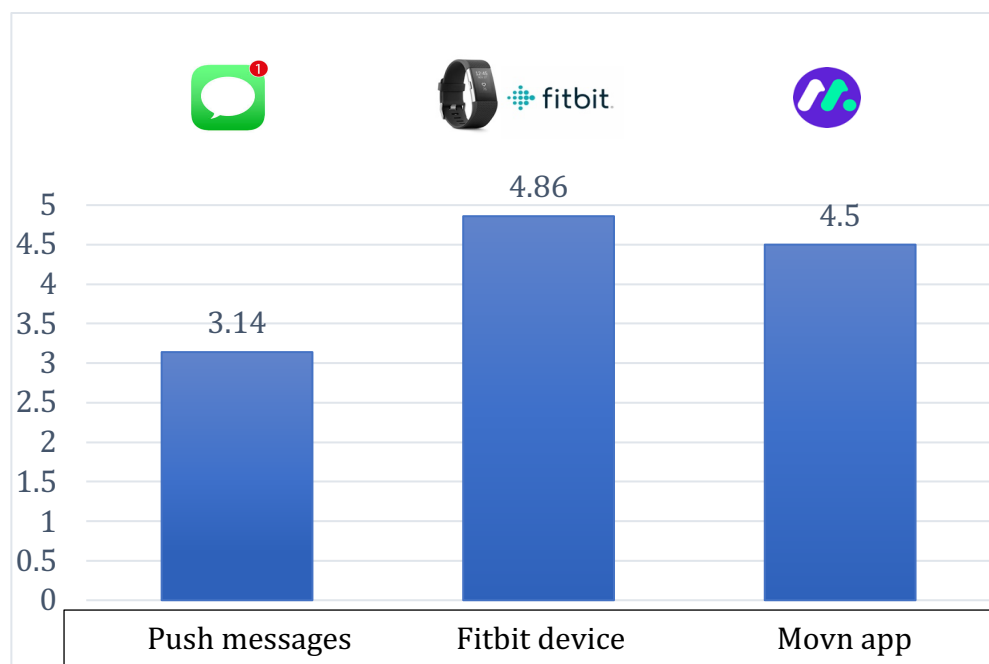

Supplement: Multimedia Appendix 4 [file humanfactors_v8i3e25356_app4.pdf]
